# The Clinical Significance of Incidental GIT Uptake on PET/CT: Radiologic, Endoscopic, and Pathologic Correlation

**DOI:** 10.3390/diagnostics13071297

**Published:** 2023-03-30

**Authors:** Mohammad N. Hosni, Mutaz Kassas, Mohamad I. Itani, Mahmoud A. Rahal, Safaa Al-Zakleet, Malak El-Jebai, Alain S. Abi-Ghanem, Hicham Moukaddam, Mohamad Haidar, Sobhan Vinjamuri, Yasser H. Shaib

**Affiliations:** 1 Division of Gastroenterology and Hepatology, Department of Internal Medicine, American University of Beirut Medical Center, Beirut 1107-2020, Lebanon; 2 Department of Diagnostic Radiology, American University of Beirut Medical Center, Beirut 1107-2020, Lebanon; 3 Department of Internal Medicine, Wayne State University School of Medicine, Detroit, MI 48202, USA; 4 Division of Gastroenterology and Hepatology, Indiana University School of Medicine, Indianapolis, IN 46202-3082, USA; 5 Nuclear Medicine, Royal Liverpool and Broadgreen University Hospital, Liverpool L7 8YE, UK

**Keywords:** positron emission tomography, gastrointestinal cancer, gastrointestinal tract, [^18^F] Fluorodeoxyglucose, endoscopy, incidental uptake

## Abstract

Incidental gastrointestinal tract (GIT) [18F]-Fluorodeoxyglucose (FDG) uptake in positron emission technology/computed tomography (PET/CT) is an unexpected and often complicated finding for clinicians. This retrospective study reviewed 8991 charts of patients who underwent PET/CT: 440 patients had incidental GIT uptake, of which 80 underwent endoscopy. Patient characteristics, imaging parameters, and endoscopic findings were studied. Of the 80 patients, 31 had cancer/pre-cancer lesions (16 carcinomas; 15 pre-malignant polyps). Compared to patients with benign/absent lesions, patients with cancer/pre-cancer lesions were significantly older (*p* = 0.01), underwent PET/CT for primary evaluation/staging of cancer (*p* = 0.03), had focal GIT uptake (*p* = 0.04), and had lower GIT uptake (*p* = 0.004). Among patients with focal uptake, an SUVmax of 9.2 had the highest sensitivity (0.76) and specificity (0.885) in detecting cancer/pre-cancerous lesions. Lower GIT uptake was most common in the sigmoid colon, and upper GIT uptake was most frequent in the stomach. In a bivariate analysis, predictors of cancer/pre-cancer were older age, PET/CT indicated for primary evaluation, focal uptake, uptake in the lower GIT, and higher SUVmax. Further endoscopic investigation is warranted for patients with incidental GIT uptake, especially in the elderly or those presenting for primary evaluation with PET/CT, with the following findings on imaging: lower GIT uptake, focal uptake, or high SUVmax.

## 1. Introduction

Positron emission tomography/computed tomography (PET/CT) is a widely used nuclear imaging technique in oncology for the detection of neoplasia [1]. [^18^F]-Fluorodeoxyglucose (FDG), the most commonly used radiolabeled tracer in PET scans, is taken up by various tissues in the human body through glucose metabolism.

The concept behind FDG PET/CT scans in the detection of malignancy is based on the observation that cancer cells metabolize glucose at a higher rate than non-cancerous cells. Hypermetabolic conditions such as infection, inflammation, and hyperplasia, as seen in colorectal polyps, may lead to increased FDG uptake [2,3]. Other physiologic reasons include uptake by microbiome or normal lymphoid tissue and swallowing of secretions [2]. Accordingly, it may be difficult to differentiate between physiologic activity and malignancy on PET/CT, and certain techniques, such as dual time point imaging, have been suggested to improve the detection of malignancy [4]. 

In the last two decades, the indications for PET/CT have widely developed in the realm of gastrointestinal cancers. Esophageal, gastric, and colorectal cancer patients can benefit greatly from the added value of PET/CT in management, prognosis, assessment of treatment response, planning of therapy, and re-staging [5]. As a result of increased PET/CT utilization in the management of cancer patients and the rising proportion of the elderly population, the incidental detection of clinically significant findings such as unexpected tumor sites has increased [6,7]. Such findings usually present a diagnostic and therapeutic challenge for clinicians. 

Multiple studies have examined incidental FDG uptake in the gastrointestinal tract (GIT) and have found variable incidence rates ranging from 0.5% to 2.6% [8,9,10,11,12,13]. A focal pattern of incidental FDG uptake in the GIT is reported to be associated with a higher likelihood of malignancy than a non-focal pattern [12,14,15]. However, these could vary depending on the indication, examined population, and the definition of abnormal uptake. The main objective of this study was to characterize the pattern and degree of incidental GIT uptake most likely to be associated with cancerous or pre-cancerous lesions in patients who underwent whole-body PET for other purposes.

## 2. Materials and Methods

### 2.1. Patient Chart Review

The medical records of patients who underwent PET/CT from January 2013 to January 2017 at a tertiary referral center and the American University of Beirut Medical Center (AUBMC) were retrospectively reviewed. All the records were initially screened for positive incidental uptake in the imaging report by a single investigator with more than 10 years of experience in nuclear medicine. For positive cases, the physician then examined the relevant PET/CT images. An incidental finding was defined as an unexpected area of increased FDG uptake in the GIT with a notable corresponding abnormality in CT images. Upper GIT uptake was defined as any uptake in the esophagus, stomach, and small intestine; lower GIT uptake was defined as any uptake in the GIT extending from the terminal ileum to the anus; intestinal uptake was defined as any GIT uptake detected exclusively in the jejunum and/or ileum; and mixed uptake was defined as any uptake occurring in both the upper and lower GIT. An incidental uptake was considered to be focal if a focal pattern was described in the radiology report. A diffuse uptake was defined as an area of increased FDG uptake along a continuous segment of the GIT. Patients were included if they were 18 years of age or older and had a positive incidental GIT finding on PET; subjects were excluded if they had a pre-existing known gastrointestinal malignancy. Data collected included age, gender, indication for PET, dose and blood glucose level, primary malignancy and type, presence of metastasis, location of incidental GI finding, pattern of FDG uptake, and endoscopic findings, if any. The study was approved by the Institutional Review Board at the American University of Beirut.

### 2.2. FDG PET/CT Imaging

Patients were instructed to fast for at least 6 h before injection of FDG except for glucose-free oral hydration. Diabetics were advised to stop oral hypoglycemic medications that contain metformin 48 h before the scan, as per the European Association of Nuclear Medicine (EANM) guidelines [16]. Blood glucose levels were measured before injection, and the PET/CT scan was rescheduled for patients with a blood glucose level >200 mg/dL. The injected dose ranged between 185 and 296 MBq. After injection, patients were kept lying comfortably. The whole body FDG PET/CT was performed using a Philips Gemini TF 16 PET/CT 60 min after injection. The CT was initially acquired for attenuation correction and anatomical correlation followed by the PET study. After completion of the PET acquisition, images were attenuation-corrected then fused with the CT images. A team of highly qualified nuclear medicine physicians with at least 10 years of experience interpreted the FDG PET/CT images using a dedicated software IntelliSpace Portal 8.0 by Philips Healthcare (Amsterdam, The Netherlands) for the revision of PET, CT, and fused PET/CT imaging data in maximal intensity projection (MIP), and axial, coronal, and sagittal planes.

### 2.3. Outcomes and Statistical Analysis

Our primary objective was to characterize the pattern and degree of incidental GIT uptake most likely to be associated with cancerous or pre-cancerous lesions in patients who underwent whole-body PET for other purposes. The statistical analysis consisted of descriptive and inferential statistics. Data were described as number and percent for categorical variables, whereas the mean and standard deviation (±SD) were calculated for continuous variables. The association between finding a cancer/pre-cancer lesion and other categorical variables was assessed using the Chi-square test, whereas Student’s t-test was used for the association with continuous variables. A bivariate analysis was performed to assess the association between finding a cancer/pre-cancer lesion and different predictors. The results included hazard ratios and 95% confidence intervals (CIs). A *p*-value < 0.05 was considered statistically significant.

## 3. Results

### 3.1. Characteristics of Studied Group

**Figure 1:**
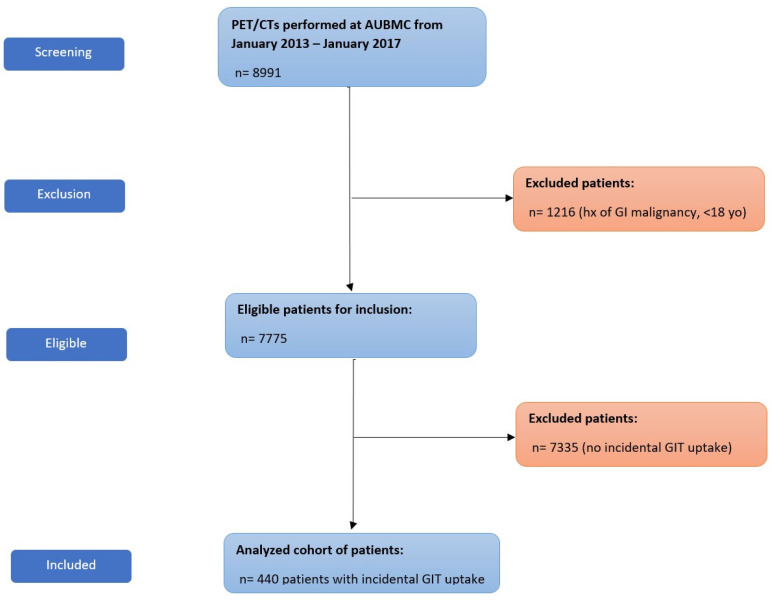
Flow diagram of patient recruitment. Abbreviations: hx, history; GI, gastrointestinal; yo, years-old.

During the study period, we screened a total of 8991 PET/CTs that were performed at AUBMC. Of those, 7775 met the inclusion criteria and 1216 were excluded because they had a history of GI malignancy, as seen in Figure 1. Incidental PET/CT uptake in the GIT was present in 440 cases. 

The general characteristics of all 440 patients are presented in Table 1. The median age of patients was 57.5 ± 16 years distributed equally between males and females. The pattern of uptake was focal in 226 cases (51%), diffuse in 199 cases (45%), and unspecified in 15 cases (3%). The most common indication for PET/CT was primary evaluation (55%). The most common types of primary malignancies were as follows: 90 cases (20.5%) of breast carcinoma, 72 cases (16.5%) of lung carcinoma, 44 cases (10%) of non-Hodgkin’s lymphoma, and 36 cases (8.2%) of Hodgkin’s lymphoma.

The general characteristics of endoscopically followed-up patients are presented in Table 2. Among the 440 cases, 80 had endoscopic evaluations. In this group, the average age of evaluated patients was 59 ± 16 years distributed equally between males and females. Most of these showed focal uptake (*n* = 57, 71%), twenty cases (25%) showed diffuse uptake, and the pattern of uptake was not specified in three cases (4%). Similar to the whole group, the most common indication for PET/CT scans was for the primary evaluation or staging of cancer (*n* = 50, 62.5%). Non-Hodgkin’s lymphoma and lung cancer (*n* = 15, 18.8%) were the most common primaries, followed by breast cancer (*n* = 13, 16.3%). The investigation of patients with an unknown primary tumor constituted 12.5% (10 cases) of endoscopically followed-up patients. 

### 3.2. Location of Uptake

**Figure 2:**
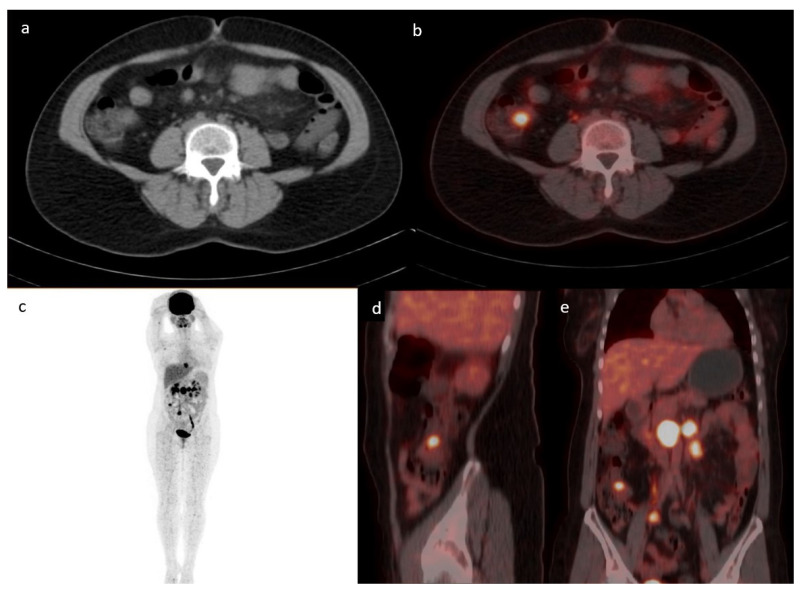
Case of a 43-year-old female with a history of follicular lymphoma. Axial CT (**a**) fused with PET (**b**) showing a focus of increased uptake at the level of the ileocecal valve with an SUVmax of 7.1 corresponding to a 1.7 cm soft tissue density. MIP (**c**) showing multiple active supra and infra diaphragmatic lymph nodes. The infra diaphragmatic lymph nodes are seen again in the fused sagittal (**d**) and coronal (**e**)-fused PET/CT images. The patient underwent a colonoscopy where ileal biopsy showed non-necrotizing granulomas, with no evidence of lymphoma or microorganisms.

The most common location for incidental GIT uptake was in the upper GIT (*n* = 212; 48%) followed by the lower GIT (*n* = 147; 33%), with the rest showing an intestinal or mixed pattern of uptake. One example of lower GIT uptake is presented in Figure 2. The location of upper GIT uptake was in the following order: stomach (174/340; 51%), esophagus (97/340; 29%), and small intestine (69/340; 20%), with 38% (128/340) of the uptake showing multiple localizations in the upper GIT. The location of lower GIT uptake was in the following frequencies: sigmoid colon (77/194; 40%), ascending colon (49/194; 25%), transverse colon (36/194; 18%), descending colon (33/194; 17%), and rectum (1/194), with 25% (47/194) of the uptake showing multiple localizations in the lower GIT. 

### 3.3. Endoscopy and Histopathology Results

Among the 80 cases, 43 patients (53.8%) underwent a colonoscopy while 37 (46.3%) underwent an upper endoscopy. Endoscopic abnormalities were present in 35 cases and were divided into polyps (*n* = 17, 49%), infiltrating mass or tumor (*n* = 13, 37%), ulcers (*n* = 4, 5%), and one stricture (3%). A total of 31 cases had a cancer/pre-cancer lesion. Twenty-nine (85%) out of the thirty-five abnormal endoscopic lesions were found to have focal incidental GIT uptake. Among the neoplastic polyps (*n* = 15), which were defined as polyps in the GIT with the potential for malignant transformation, tubular adenomas were most commonly present (*n* = 11, 71%), followed by tubulo-villous (*n* = 3, 21%), and villous (*n* = 1, 7%) adenomas, as seen in Table 3.

Out of the pathology-confirmed lesions, twenty-six exhibited a focal uptake, whereas only five showed a diffuse uptake. Lesions displaying a diffuse uptake on FDG PET/CT are further characterized in Table 4.

**Figure 3:**
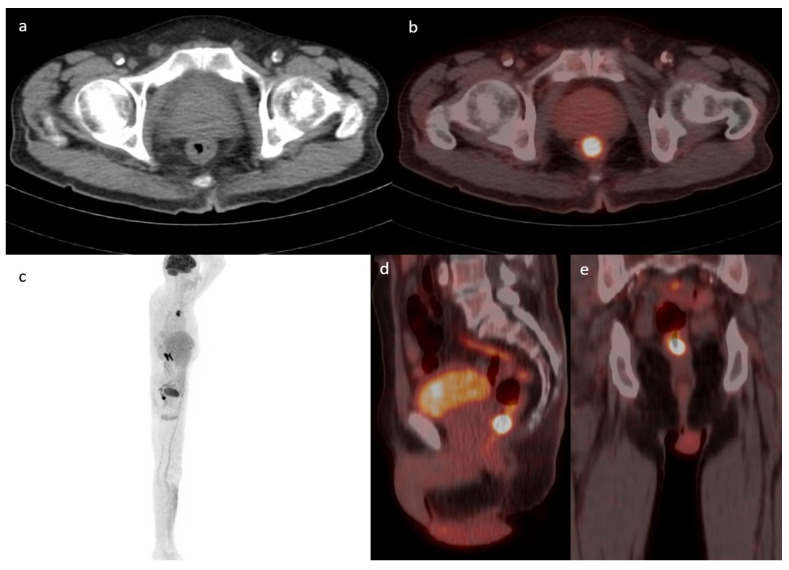
Case of a 71-year-old male known to have primary bladder cancer. Axial cuts of CT (**a**) fused with PET (**b**) showing a focus of increased uptake in the lower rectum (SUVmax 19.1) with corresponding circumferential wall thickening suspicious for malignancy. This lesion is redemonstrated in the MIP (**c**), sagittal (**d**), and coronal PET/CT cuts (**e**). Rectal biopsy confirmed the diagnosis of moderately differentiated adenocarcinoma.

On pathologic examination, of the sixteen detected carcinomas, twelve were biopsied from a mass while the other four were detected on pathology (absence of a mass on endoscopy). Of the sixteen detected carcinomas, seven were in the stomach and one was detected in the jejunum while the remaining eight were present in the colon. One example is presented in Figure 3. Patients with incidental upper GIT malignancy were younger than the studied population (53 ± 17.6 compared to 64.6 ± 16 years). All malignancies in the lower GIT showed focal uptake, while 5/8 (62.5%) in the upper GIT had focal uptake. The remaining 3/8 (37.5%) malignancies were detected as diffuse uptake in the upper GIT. The characteristics of all detected cancer/pre-cancer lesions are summarized in Table 5 and Table 6.

### 3.4. Statistical Analysis

In the bivariate analysis presented in Table 6, patients with cancer/pre-cancer lesions (*n* = 31) (pre-cancerous lesions and carcinomas) were significantly older (mean age = 65 years ± 16, *p* = 0.011), more likely to undergo a PET/CT scan for primary evaluation (*p* = 0.028), and more likely to have focal GIT uptake (*p* = 0.043) and lower GIT incidental uptake (*p* = 0.004) when compared to those with benign lesions (*n* = 49). Among patients with a focal GIT uptake, patients who had a cancer/pre-cancer lesion were significantly older (*p* = 0.02), were more likely to have a lower GIT uptake (*p* = 0.001), and had a higher maximum standardized uptake value (SUVmax) (*p* = 0.001).

The corresponding percentages of benign versus pre-malignant/malignant lesions were observed in SUVmax strata of 3–5, >5–7, >7–10, >10–14, >14–20, and >20, in the respective order: 75% vs. 25%, 69% vs. 31%, 65% vs. 35%, 22% vs. 78%, 20% vs. 80%, and 0% vs. 100% in the final group, as seen in Table 7.

**Figure 4:**
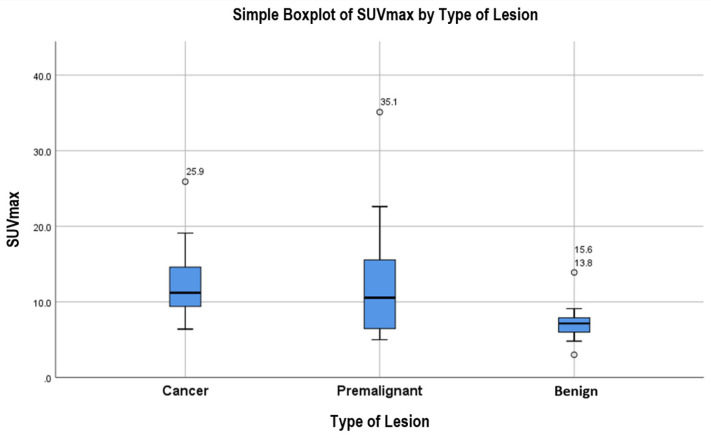
Simple boxplot of SUVmax comparison between benign, pre-malignant, and malignant lesions.

**Figure 5:**
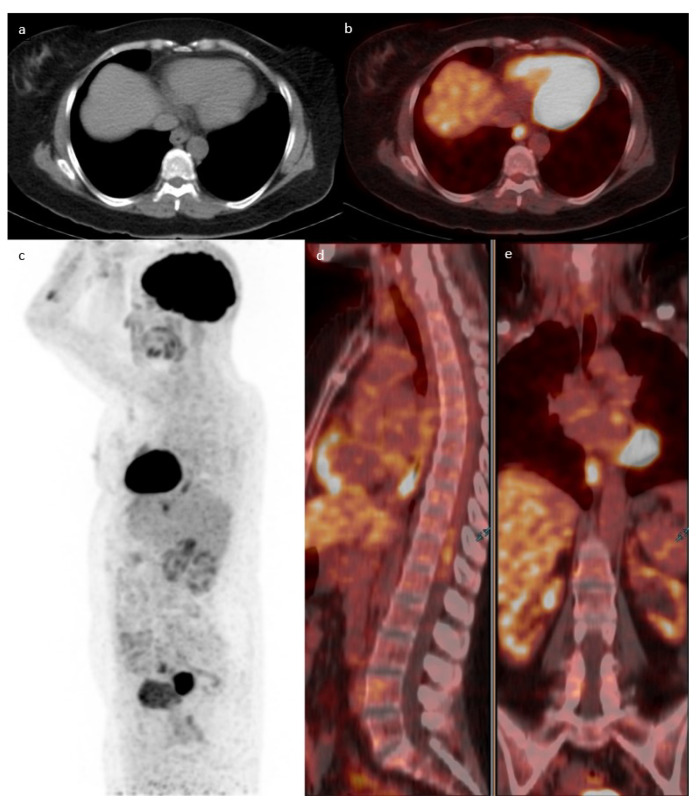
Case of a 70-year-old female known to have uterine cervical cancer. Axial CT (**a**) fused with PET (**b**) showing a focus of uptake in the distal esophagus with an SUVmax of 5.6. MIP (**c**)-fused sagittal (**d**) and axial PET/CT (**e**) showing increased linear uptake within the distal aspect of the esophagus. The patient underwent gastroscopy, which revealed inflammatory processes in keeping with esophagitis and no evidence of malignancy.

Figure 4 compares the different SUVmax values between three different groups (malignant, pre-malignant, and benign lesions) among patients with focal uptake, with one example of a benign finding presented in Figure 5. Patients with malignant and pre-malignant disease had higher SUVmax with a median of 11.2 (IQR = 9.4, 15.3) and 10.54 (IQR = 6.4, 17.1), respectively, compared to a median of 7.15 (IQR = 5.98, 8) for benign lesions. To assess the role of SUVmax in detecting a truly positive lesion on PET/CT, we used the receiver operating characteristic (ROC) curve. We defined sensitivity as the fraction of occurrence, and specificity as the fraction of absence of cancer/pre-cancer lesions on further investigation with endoscopy. This analysis showed a statistically significant area under the curve of 0.79 (*p* < 0.0001). An SUVmax of 9.2 had the highest sensitivity and specificity of 0.76 and 0.885, respectively.

After adjusting for confounders (age, previously known metastatic cancer, indication for undergoing PET/CT scan, location of uptake, pattern of uptake), the predictors of a cancer/pre-cancer lesion on further evaluation were as follows: older age (OR = 1.04, [1.006, 1.077]), undergoing PET/CT for primary evaluation (OR = 3.57, [1.15, 11.11]), and having incidental uptake in the lower GIT (OR = 4.69, [1.55,14.2]). Similarly, after selecting cases with a focal incidental GIT uptake (*n* = 57), the location of the uptake (lower GIT) (OR = 8.14, [1.77, 37.54]), along with a higher SUVmax (OR = 1.41, [1.10, 1.80]) were predictors of finding a cancer/pre-cancer lesion.

## 4. Discussion

Fused PET/CT has been widely used for the staging and follow-up of several types of malignancies, and this has accordingly increased the rate of incidental findings in the GIT [17]. A recent study by Byung et al. (2016) showed that FDG PET/CT had a high diagnostic accuracy for detecting synchronous colorectal cancer in patients with gastric cancer [18]. Several studies have shown that FDG uptake in the gastric mucosa could be focal or diffuse in a variety of benign conditions (normal mucosa, gastritis, H. pylori infection) [19]. Similarly, in colorectal carcinoma (CRC), a meta-analysis showed the sensitivity and specificity for PET in the detection of CRC recurrence in patients with elevated CEA to be 90% and 80%, respectively [20].

Several studies have assessed incidental FDG uptake in the GIT, but only a few included those with diffuse uptake or with upper GIT uptake. Our study is one of the largest to include any incidental GIT uptake (including upper and diffuse uptake) that was followed-up endoscopically. The rate of incidental GIT uptake in our study population was 5.6%, with 80 patients (18.2%) undergoing further investigation. Prior studies reported the incidental GIT uptake to range from 0.7 to 2.6%, with more than 50% of the patients not undergoing further endoscopic evaluation [9,10,15]. However, these studies only included focal uptake and excluded uptake in the upper GIT. In our study, 29% of all patients with an abnormal lesion on endoscopy had diffuse FDG uptake on PET, which was comparably higher than the 1–17% detected in similar studies [15,21].

Although an incidental uptake in the lower GIT has more likelihood of being a cancer/pre-cancer lesion, 50% of the malignant lesions in our study were found in the upper GIT (*n* = 8). Patients with incidental upper GIT malignancy were younger than the studied population. A study conducted by Buel et al. (2002) identified 34 solid organ recipients who were on immunosuppressive therapy and developed gastric cancer [22]. Patients had a mean age of 58 ± 11, and 22 out of the 34 detected gastric cancers were found incidentally [22]. Another study performed by Isobe et al. (2010) showed that among twenty-six cases of incidental GIT uptake on PET/CT for patients with lung cancer, four malignant lesions were found in the upper GIT [23]. In our study, 37.5% of all biopsy-proven malignancies in the upper GIT were visualized as a diffuse FDG uptake. This percentage was higher than the 23% reported in one recent study also examining diffuse uptake in the upper GIT [21].

In clinical practice, it would be of benefit for the clinician to have a general idea of the probability of malignancy based on uptake pattern and location. When the results were examined based on the percentage of disease found in focal and diffuse uptake, the following became apparent: 46% of focal uptake was due to abnormal lesions, compared to only 30% of diffuse uptake cases. Upon further classification based on location, 5/57 of all focal uptake cases were upper GIT malignancies and 8/57 were lower GIT malignancies. As for diffuse uptake, 2/20 of all diffuse uptake represented upper GIT malignancies.

Upper GIT uptake was most frequent in the stomach. One retrospective cohort by Gilhotra et al. (2022) showed the most common upper GIT uptake in the esophagus (58.4%); however, this can be explained by inflammatory changes detected in 57% of these patients [21]. As for the lower GIT uptake, it was most commonly observed in the sigmoid colon. This finding is in concordance with Gilhotra et al. (2022) on patients with incidental lower GIT uptake, where 67% of all patients had uptake in the left colon [21]. It is worth noting that this uptake is not an absolute reflection of CRC incidence because the uptake may be explained by physiologic processes; moreover, the site of the highest frequency of colorectal cancer is highly variable among different population groups [24].

Our study showed that patients who were older in age or had lower GIT uptake were more likely to have a cancer/pre-cancer lesion on further endoscopic evaluation regardless of uptake pattern. However, in patients with focal uptake, the intensity of the uptake was shown to be an independent predictor of finding a cancer/pre-cancer lesion. In our study, an SUVmax greater than 9.2 had the highest sensitivity (0.76) and specificity (0.885). In the literature, one study by Lee et al. (2022) showed a sensitivity of 0.686 and a specificity of 0.688 at an SUVmax cut-off of 7.6 for the differentiation between benign and cancer/pre-cancerous lesions, without having any significant difference in parameters when distinguishing between cancer and pre-cancerous lesions [25]. Another retrospective study by Ozaslan et al. (2021) found a significantly higher mean SUVmax in patients with malignancy (15.0) in comparison to adenomas (10.2); however, they detected a mean SUVmax of 9.8 in normal endoscopic groups [26]. This would mean that an SUVmax cut off of 9.2, shown to have a specificity of 0.885 in this study, would lead to a high number of false positives when used in their study. Accordingly, a 9.2 SUVmax cut off may not be of value when applied to other populations. In contrast to these findings, one large retrospective study by Gokden et al. (2022) showed no significant difference between the SUVmax of malignant and benign cases; however, they detected significantly higher mean SUVmax in malignant cases in comparison to positive cases that were less high-grade [27].

Although incidental FDG uptake in the GIT has a low positive predictive value (0.387), several studies have concluded that it warrants a follow-up endoscopy (esophagogastroduodenoscopy or colonoscopy) [28,29]. Many studies have assessed colonic uptake, but very few investigated diffuse or upper GIT uptake. In our study, a cancer/pre-cancer lesion was found in eight out of the thirty-seven incidental upper GIT findings (21.6%) as well as in five out of twenty diffuse uptake cases in the GIT (25.0%).

One consideration that may explain the higher incidence of lower GIT uptake may be the use of metformin-containing anti-diabetics in patients. Metformin is previously known to increase the FDG uptake in the GIT [30]. Accordingly, the EANM advises on the discontinuation of metformin two to three days before imaging [16]. In our study, patients were advised to discontinue any metformin-containing anti-diabetic medication at least 48 h before the imaging. It was not documented if the patients actually withheld from taking the medication, and this may have led to a falsely elevated incidental uptake in the lower GIT. Moreover, documentation of patient diabetic status was not included in the baseline characteristics, which may be a confounder revealing elevated rates of lower GIT uptake in diabetic patients if a subgroup analysis was performed.

One major consideration to the further investigation of all incidental FDG GIT uptake on PET would be the financial burden and time consumption in a clinic or hospital setting. One study by Hadad et al. (2020), which was performed in a European country, has shown that the estimated additional cost to investigating each focus of FDG uptake in the GIT is approximately USD 1984 [31]. The price of further investigations is highly variable, where another study by Adams et al. (2018) conducted in Canada showed an average cost of USD 127 [6].

A subgroup analysis on other patient characteristics such as BMI and co-morbidities is needed. One other limitation in this study is the single-center study design, which reduces the external validity of the findings. A prospective, multi-center study with standardized imaging parameters is needed to reduce possible confounders that may elicit heterogeneity among studies. A holistic view of the patient’s history and clinical picture is required to optimize management. The clinician’s better judgment is needed, while maintaining a low threshold for further investigation.

## 5. Conclusions

Further endoscopic investigation is warranted for patients with incidental GIT uptake, especially in the elderly or those presenting for primary evaluation with PET/CT, with the following findings on imaging: lower GIT uptake, focal uptake, or high SUVmax. More studies should investigate incidental upper GIT uptake to detect early gastric carcinomas in patients with non-GIT malignancies, and a subgroup analysis should be a cornerstone for future studies examining incidental GIT uptake.

## Figures and Tables

**Table 1:** General characteristics of all 440 patients.

General Characteristics		Number (*n* = 440)	Percentage (%)
Gender	Male	221	50
	Female	219	50
Indication for PET/CT	Primary evaluation/staging	200	55
	Follow-up	240	45
Type of Primary	Breast carcinoma	90	20.5
	Lung carcinoma	72	16.5
	Non-Hodgkin’s lymphoma	44	10
	Hodgkin’s lymphoma	36	8.2
	Other ^1^	198	44.8
FDG Uptake	Focal	226	51
	Diffuse	199	45
	Unspecified	15	3
Location of Uptake	Upper	212	48
	Lower	147	33
	Intestinal	41	9
	Mixed	40	9
Regional or Distant Metastasis from Primary	Absent	265	60
	Present	175	40

^1^ Other includes adrenal carcinoma, bladder carcinoma, brain tumor, cervical cancer, endometrial carcinoma, fever of unknown origin, unknown primary, multiple myeloma, nasopharyngeal carcinoma, and ovarian carcinoma.

**Table 2:** General characteristics of the endoscopically followed-up population.

General Characteristics		Number (*n* = 80)	Percentage (%)
Gender	Male	39	49.8
	Female	41	50.2
Indication for PET/CT	Primary evaluation/staging	50	62.5
	Follow-up	30	37.5
Type of Primary	Lung carcinoma	15	18.7
	Non-Hodgkin’s lymphoma	15	18.7
	Breast carcinoma	13	16.3
	Unknown primary	10	12.5
	Other ^1^	27	33.8
FDG Uptake	Focal	57	71
	Diffuse	20	25
	Unspecified	3	4
Location of Uptake	Upper	27	34
	Lower	34	42
	Intestinal	8	10
	Mixed	11	14
Biopsies	Obtained	63	78.8
	Not obtained	17	21.2

^1^ Other includes adrenal carcinoma, bladder carcinoma, brain tumor, cervical cancer, endometrial carcinoma, fever of unknown origin, Hodgkin’s lymphoma, multiple myeloma, nasopharyngeal carcinoma, and ovarian carcinoma.

**Table 3:** Pre-malignant and malignant lesions found upon endoscopic investigation.

Pre-Malignant Polyps (15)	*n* (%)
Tubular adenoma	11 (71%)
Tubulovillous adenoma	3 (21%)
Villous adenoma	1 (7%)
**Cancerous Lesions (*n* = 16)**	***n* (%)**
Moderately differentiated adenocarcinoma	9 (56.3%)
Poorly differentiated adenocarcinoma	3 (18.8%)
Lymphoma	3 (18.8%)
Adenocarcinoma of breast origin	1 (6.3%)

**Table 4:** Characteristics of the cancerous or pre-cancerous lesions in patients with diffuse FDG uptake.

Patient	Location of Diffuse Uptake	Pathology Findings
1	Stomach	Metastatic adenocarcinoma from breast primary
2	Stomach	Intestinal metaplasia
3	Jejunum	Diffuse large B-cell lymphoma
4 *	Descending Colon	Colon adenocarcinoma primary
5 *	Sigmoid Colon	Diffuse large B-cell lymphoma

* These patients had mixed uptake patterns and were followed-up with endoscopy.

**Table 5:** Clinical characteristics of incidental gastrointestinal tract (GIT) malignancies in the upper and lower GIT.

Characteristics	Upper Incidental Gastrointestinal Tract Malignancy (*n* = 8)	Lower Incidental Gastrointestinal Tract Malignancy (*n* = 8)
Gender (male), *n* (%)	4 (50%)	4 (50%)
Age, mean ± SD	53 ± 17.6	66.7 ± 18.9
Metastatic disease, *n* (%)	6 (75.0%)	4 (50.0%)
Indication (primary evaluation)	6 (75.0%)	8 (100.0%)
Focal ^18^FDG GIT uptake, *n* (%)	5 (62.5%)	8 (100.0%)
SUVmax, mean ± SD	10.7 ± 3.5	14 ± 5.8

**Table 6:** Bivariate analysis comparing the clinical characteristics of patients with benign and malignant findings upon endoscopic evaluation.

Variable	Cancer/Pre-Cancer Lesions (31)	Benign Findings (49)	*p*-Value
Age (mean ± SD)	64.6 ± 16	55.1 ± 16	0.011
Gender (male)	17 (54.8%)	22 (44.9%)	0.49
Focal uptake	26 (86.7%)	31 (66.0%)	0.043
Metastatic cancer	14 (34.1%)	27 (55.1%)	0.39
Indication (primary evaluation)	24 (77.4%)	26 (53.1%)	0.028
Location of uptake (lower)	23 (74.2%)	20 (40.8%)	0.004
**Among Patients with Focal Uptake (57)**			
Age (mean ± SD)	66.4 ± 15.7	56.1 ± 16.8	0.02
Gender (male)	14 (53.8%)	13 (41.9%)	0.37
Metastatic cancer	11 (42.3%)	18 (58.1%)	0.236
Indication (primary evaluation)	20 (76.9%)	18 (58.1%)	0.132
Location of uptake (lower)	21 (80.8%)	11 (35.4%)	0.001
SUVmax (mean ± SD)	12.8 ± 7.0	7.6 ± 2.8	0.001

**Table 7:** Percentage of benign and pre-malignant/malignant lesions in each SUVmax group.

SUVmax Range	Benign	Pre-Malignant/Malignant
3–5	75%	25%
>5–7	69%	31%
>7–10	65%	35%
>10–14	22%	78%
>14–20	20%	80%
>20	0%	100%

## Data Availability

Data will be available upon request from the corresponding author.
